# Long-Term Consequences of Nonclosure of Mesenteric Defects after Traditional Right Colectomy

**DOI:** 10.1155/2018/9123912

**Published:** 2018-09-26

**Authors:** Kai-Lung Tsai, Wei-Hung Lai, Ko-Chao Lee, Shung-Eing Lin, Chia-Lo Chang, Chien-Chang Lu, Wan-Hsiang Hu, Seng-Kee Chuah, Hong-Hwa Chen

**Affiliations:** ^1^Division of Colon and Rectal Surgery, Department of Surgery, Kaohsiung Chang Gung Memorial Hospital, 123 Ta-Pei Road, Niao-Song District, Kaohsiung 833, Taiwan; ^2^Department of Trauma Surgery, Kaohsiung Chang Gung Memorial Hospital, 123 Ta-Pei Road, Niao-Song District, Kaohsiung 833, Taiwan; ^3^Division of Gastroenterology, Kaohsiung Chang Gung Memorial Hospital, College of Medicine, Chang Gung University, 833 Kaohsiung, Taiwan

## Abstract

**Background:**

There are still discrepancies among general/colorectal surgeons regarding closure of mesenteric defect in scientific literature. This study aimed to assess the long-term consequences of nonclosure of the mesenteric defect after open right colectomy.

**Methods:**

A 7-year retrospectively collected and continuous database revealed 212 consecutive patients who had undergone traditional right colectomy without closing the mesenteric defects at Kaohsiung Chung-Gung Memorial Hospital; all patients were operated by a single surgeon. Among these patients, 17 were excluded (those who died within 30 days after surgery or those who received an end ileostomy). The mean age of the 195 patients (58% men and 42% women) was 61.6 ± 12.6 years, and the follow-up period was 4.1 ± 2.8 years (interquartile range 0.09 ~ 10.4).

**Results:**

Forty-four patients (22.5%) encountered intestinal obstruction. Nine (20.4%) required surgical intervention. The cause of intestinal obstruction was adhesion (n=1), ventral hernia (n=1), and cancer recurrence (n=7). Conservative treatment was successful in 35 patients. The intestinal obstruction group (n = 44) were similar to the no-intestinal obstruction group (n = 151) in terms of the following parameters: age, sex, previous abdominal surgery, indication for colectomy, and procedure related complications. Carcinomatosis was found to increase the incidence of intestinal obstruction. No patient developed intestinal obstruction because of the nonclosure of mesenteric defects after right colectomy.

**Conclusion:**

This study suggested that routine procedure of closing the mesenteric defect after open right colectomy might not be beneficial. Additional studies with extended long-term follow-up periods are needed to confirm the benefits of the nonclosure.

## 1. Introduction

Intestinal obstruction is an uncommon complication of open mesenteric defects and, due to internal herniation, is even rarer [1–4]. Nonetheless, intestinal obstruction as a complication should always be kept in mind. Nonclosure of the mesenteric defect may cause internal herniation, which in turn can cause intestinal obstruction [5–9]. However, the occurrence of such obstruction has not been well documented, and closure of mesenteric defects remains in debate [10–12]. In the past, there were case reports on the issues regarding repair of the mesenteric defect of right colectomy as the standard procedure [13–15]. However, large-scale studies investigating and comparing the consequences of repair or nonclosure of the mesenteric defect during operation are scarce. This study aimed to assess the long-term consequences of nonclosure of the mesenteric defect after open right colectomy.

## 2. Patients and Methods

### 2.1. Ethics Statement

The study protocol was approved by the institutional review board and the Ethics Committee of Chang Gung Memorial Hospital, Taiwan. The Ethics Committee waived the requirement for informed consent for this study, and all the data were analyzed anonymously.

### 2.2. Patients

A total of 212 patients had undergone right side colectomy for the treatment of various conditions at Chang Gung Memorial Hospital in Kaohsiung. Patient data were obtained from a 7-year, retrospectively collected, continuous, single-institution database, which included data of patients who underwent elective or emergent right colon resection; all patients were operated by a single surgeon. The sources of the collected data were questionnaires survey, systematic review of charts, office records, radiographic imaging data, and patient interviews. In our institute, the presence of intestinal obstruction is mainly determined on the basis of clinical presentation and patient complaints; for example, the absence of passage of flatus and/or feces and vomiting are the most common presenting symptoms.

The operative reports were studied to determine the technical aspects with regard to the mesenteric defect; all reports documented whether the defect was closed. Five patients who died within the first postoperative month and twelve patients who underwent resection with end ileostomy were excluded from this study. Therefore, the study population comprised the remaining 195 patients. The following information was recorded for each patient: age, sex, intraoperative and postoperative courses, incidence and treatment of intestinal obstruction, and follow-up period. Information regarding the incidence of intestinal obstruction was gathered from office charts and hospital records. Intestinal obstruction was determined by clinical assessment, operative findings, and imaging findings.

### 2.3. Surgical Techniques of Right Colectomy

A liberal midline incision centered about the umbilicus was made to start the operation and the incision was carried down into abdominal cavity layer by layer. The wound was protected with wet pads and the retractor was put to provide the optimal operation view. The position of the nasogastric tube was checked to assure its appropriate site. Liver, peritoneum, and entire bowel were inspected and palpated. The small bowel was walled off with warm pads. An incision was then made in the peritoneal reflection close to the lateral wall of the bowel from the tip of the cecum upward to the region of the hepatic flexure. The hepatocolic ligament was divided and ligated. With the lateral peritoneal attachment divided, the large bowel was lifted with hand and the loose areolar tissue lying under lifted large bowel was dissected off with a moist gauze sponge. After we made sure the gonadal vessels, the ureter, and the secondary part of duodenum were visualized, the dissection distal to hepatic flexure was continued by entering the lesser sac to avoid injury to these structures. The stomach was then grasped and put on traction by pulling the omentum downward so that the greater omentum could be divided beyond the gastroepiploic arcade to the point at which the bowel could be divided. This part of the mobilization was continued approximately to the proximal third of the transverse colon. At this point, the remaining raw surface of the right colon was freed to be brought outside the peritoneal cavity and covered with warm, moist gauze pads.

The mesentery was then divided by transillumination so that the main vessels such as ileocolic, right colic and right branch of middle colic artery could be ligated as near to their origin as possible. The mesenteric division was continued to the point on the ileum at which it was intended to be resected at approximately 5 cm proximal to the ileocecal valve. Subsequently, the mesentery was divided close to the transverse colon in order to securely ligate the marginal vessel between the two branches of the middle colic artery.

Division of terminal ileum and transverse colon was performed by using linear staple. Then the lesion was successfully removed and closure of both bowel ends achieved. Transverse incisions were made near the ends of terminal ileum and transverse colon to ensure a matched length was achieved. The contents of the open ileum and transverse colon were then swabbed with 40% alcohol. After aligning the above two incisions, full-thickness suture through the posterior walls was then performed by tying on the inner side of the anastomosis. Then we proceeded with another continuous suture for the anterior wall by the same method plus second layers interrupted seromuscular sutures. A final careful check for bleeding ligated vessels and cauterization was applied if it occurred. Eventually, a drain tube was inserted to the subphrenic space. Small bowel was put back in its anatomic position and covered with omentum. The surgery ended by continuous sutures approximate to the peritoneum and linea alba from the upper end of the incision downward followed by skin closure.

### 2.4. Statistical Analysis

The data were statistically analyzed using the Chi-square test. All statistical analyses were conducted using the statistical software package SPSS (IBM Corp. Released 2012. IBM SPSS Statistics for Windows, Version 21.0. Armonk, NY: IBM Corp.) A P value of <0.05 was considered statistically significant. The P value was not adjusted for multiple tests.

## 3. Results

All the 195 patients had undergone right colectomy with nonclosure of the mesenteric defect and without use of adhesion barriers. The baseline data of these patients were demonstrated in Table 1. Of these, 113 (58%) were men and 82(42%) women, and the mean age was 61.6 ± 12.6 years. The indications for right colectomy were as follows: cancer (80%), benign tumor or polyp (7.7%), or other conditions including diverticulitis or appendicitis (12.3%). A total of 45 patients (23%) had a history of abdominal surgery for other indications. The median follow-up period was 4.23 years (interquartile range, 0.09–10.35). The office charts and hospital records of all of the patients were reviewed. The details of the complication reported in the 16 patients (8%) were summarized in Table 2. Ten of the 16 patients developed wound infection and all of them were cured by wound care and antibiotics. Two patients had postoperative chyloperitoneum and gradually recovered after diet education and total parenteral nutrition support. One patient encountered delayed gastric emptying and was initially treated by nasogastric tube decompression and was recovered eventually. There were only two patients who required additional surgical interventions. One of them suffered from anastomosis leakage and received Hartmann's operation. The other one experienced wound dehiscence and received surgical repair. Both of them recovered from the second surgical treatment. There was only one mortality in this study which occurred in an 88-year-old male patient with ascending colon cancer who expired due to sepsis.

Intestinal obstruction was detected in 44 patients (22.6%) during the follow-up; these patients were admitted to our emergency room for treatment of abdominal distension, abdominal cramps, nausea, and vomiting; 18 patients were discharged after the symptoms subsided, 17 patients were admitted for further conservative treatment, and 9 patients required surgical intervention. Intestinal obstruction developed in 3 patients within the first postoperative month in 14 patients within the first postoperative year. The longest interval between right colectomy and the occurrence of intestinal obstruction was 4.73 years. Thirty-five patients successfully responded well to conventional nonoperative treatment. Their symptoms subsided about 2 days of admission in our ward, and the mean hospital stay was about 4 days. Nine patients either did not respond to nonoperative therapies and therefore required surgery or primarily require urgent surgical intervention. The causes of obstruction in this group were adhesion (1 patient), ventral hernia (1 patient), and recurrent tumor (7 patients) (Table 3). Among the patients with intestinal obstruction, a significantly higher percentage of patients had carcinomatosis. No statistically significant differences were found in any of the other parameters examined between the groups of patients with and without intestinal obstruction (Table 4).

## 4. Discussion

Intestinal obstruction is diagnosed mainly on the basis of clinical manifestations rather than on laboratory data or imaging findings; therefore we determined cases of intestinal obstruction by using the data from chart records and the patient's description [16, 17]. Intestinal obstruction was considered present if the patient visited our hospital complaining of abdominal cramps, abdominal distension, nausea, and vomiting. The high percentage of cases of intestinal obstruction in our study series may be because, in our hospital, we admitted patients with intestinal obstruction if they stayed in the emergency room for > 1 day or if the time predicted for the symptoms to subside was > 1 day.

Internal hernia of the small bowel through a mesenteric defect following colorectal cancer surgery is a serious complication with limited reports in the literature. Lee reported 0.2% patients who presented with symptomatic internal hernia after surgical procedures such as laparoscopic anterior resection which included low anterior resection and intersphincteric resection [18]. Although laparoscopic colorectal surgery create fewer adhesion compared with open colorectal resection, small bowel obstruction may still be caused by internal herniation of the small bowel through a colonic mesenteric defect, probably related to a lack of adhesion formation. This is seen especially after left colonic resections. It is still in debate whether nonclosure of the mesenteric defect increases internal herniation, which in turn can cause intestinal obstruction. Cabot et al. reported a 7-year database of 530 consecutive patients who underwent laparoscopic right colectomy for neoplasia without mesenteric defects [10]. The data did not support routinely closing the mesenteric defect after laparoscopic right colectomy for neoplasia. Kim reported that early postoperative small bowel obstruction following laparoscopic resection for colorectal cancer occurred in 5.9% of patients [19]. In our study, most patients with early postoperative small bowel obstruction improved with conservative treatment, and surgical treatment was rarely needed. On the other hand, Sugiyama et al. supported the closure of mesenteric defects after laparoscopic right colectomy because serious complications requiring reoperation occurred only in the nonclosure group [20]. Moreover, the procedure for closing the defect did not extend the operation time or increase the bleeding. In addition, there were other case reports indicating serious complications as a result of internal hernia and subsequent obstruction [7, 21].

From our experience, nonclosure of the mesenteric defects caused abdominal distension, cramps, nausea, and vomiting in most patients, and symptoms subside after conservative treatment; only some of the patients required hospitalization for close observation, and only a few patients required surgical intervention. This was quite similar to the report of Cabot et al. In our study, the only parameter that affected the incidence of intestinal obstruction was carcinomatosis, which was the expected outcome. Moreover, mesenteric defects were not associated with a significant incidence of clinically relevant internal herniation. Forty-four out of the 195 patient had symptoms/signs of intestinal obstruction, but internal herniation or adhesion to the mesenteric defect was not evident in any of these patients. These data support the practice of not closing the mesenteric defect after right colectomy performed for any condition.

With respect to the limitation of our study, apart from the inherent shortcomings of a retrospective study, study was limited by our inability to accurately determine the cause of intestinal obstruction in patients who responded well to conservative treatment or in asymptomatic patients. Therefore, the incidence of internal herniation may have been underestimated in these patients. The results of the study on laparoscopic right colectomy for colon cancer without mesenteric repair do not support the routine closure of the mesenteric defect after laparoscopic right colectomy [10]. Importantly, in these studies, the mean follow-up period was < 2 years whereas in our study, the mean follow-up period was 4.1 years. In our study, it was because many patients were diagnosed at the advanced malignancy stage (carcinomatosis) that could affect the mean follow-up time. Therefore, studies with extended long-term follow-up periods are needed to further evaluate the practice of whether or not to close the mesenteric defect in right colectomy.

## 5. Conclusion

This study suggested that the routine procedure of closing the mesenteric defect after open right colectomy might not be beneficial. Additional studies with an extended long-term follow-up period are still needed to confirm the benefits of the nonclosure, as literature has numerous small series.

## Figures and Tables

**Table 1:** Summary of patients' demographic data.

Variables	
Age, years	62.9 ± 13.2
Sex, male : female (No, %)	113:82 (58:42)
Previous abdominal surgery (No, %)	45 (23)
Complications (No, %)	16 (8)
Indication for surgery (No, %)	
Cancer (No, %)	156 (80)
Benign tumor (No, %)	15 (7.7)
Others (No, %)	24 (12.3)
Surgery for intestinal obstruction (No, %)	9 (4.6)
Intestinal obstruction (No, %)	44 (22.6)
Visit emergency department (No, %)	18 (41)
Admission (No, %)	17 (38.6)
Operation (No, %)	9 (29.4)
Intestinal obstruction due to mesenteric defect (No, %)	0
Mean follow-up time, years	4.1 ± 2.8

**Table 2:** Postoperative complications of the patients.

Patients	Clinical manifestations	Age (years) /Sex	Location of the lesion	Underlying diseases	Therapy	Outcome
1	Wound infection	76/Male	Cecal cancer	Pulmonary tuberculosis	Wound care Antibiotics	Recovery
2	Delay gastric empty	82/Male	Hepatic flexure colon cancer	Diabetes mellitus, hypertension, prostate cancer	Nasogastric tube decompression and total parenteral nutrition support	Recovery
3	Wound infection	52/Male	Hepatic flexure colon cancer	Liver cirrhosis	Wound care Antibiotics	Recovery
4	Respiratory failure, sepsis, intra-abdominal abscess	88/Male	Ascending colon cancer	Cardiac arrhythmia	Antibiotics	Expired
5	Chyloperitoneum	83/Male	Hepatic flexure colon cancer	Liver cirrhosis, moderate atrial regurgitation	Diet education and total parenteral nutrition support	Recovery
6	Chyloperitoneum	66/Male	Hepatic flexure colon cancer	None	Diet education and total parenteral nutrition support	Recovery
7	Wound infection	63/Female	Transverse colon cancer	Gastric ulcer Diabetes mellitus	Surgical debridement	Recovery
8	Wound infection	70/Female	Cecal cancer	None	Wound care Antibiotics	Recovery
9	Anastomosis leakage	77/Female	Hepatic flexure colonic cancer	Gall stone	Hartmann's operation	Recovery
10	Wound infection	32/Male	Appendix lymphoma	None	Wound care Antibiotics	Recovery
11	Wound infection	66/Male	Cecal cancer	None	Wound care Antibiotics	Recovery
12	Wound infection	70/Male	Ascending colon polyp	None	Wound care Antibiotics	Recovery
13	Wound dehiscence	74/Male	Cecal tumor	Benign prostate hypertrophy	Surgical repair	Recovery
14	Wound infection	51/Male	Ascending colon ischemic colitis	Diabetes mellitus	Wound care Antibiotics	Recovery
15	Wound infection	47/Female	Rupture appendicitis	None	Wound care Antibiotics	Recovery
16	Wound infection	41/Male	Cecocutaneous fistula	Status post appendectomy	Wound care Antibiotics	Recovery

**Table 3:** Intestinal obstruction characteristics.

Total no. of cases	44 (22.6%)
No. of surgically treated	9 (20.4%)
Etiology	
Adhesion	1 (11.1%)
Ventral hernia	1 (11.1%)
Cancer recurrence	7 (77.8%)

**Table 4:** Comparison of patient groups.

	Patients with Intestinal obstruction (n = 44 patients)	Patients with no intestinal obstruction (n = 151 patients)	***p-value***
Age, y	62.1 ± 12.81	63.1 ± 13.4	0.663
Sex (male)	22	22	0.225
Previous abdominal surgery	13	32	0.247
Peritoneal seeding	20	35	0.004
Complication	3	13	0.703
Indication for surgery			
Cancer	36	120	0.732
Benign tumor	1	14	0.125
Others	7	17	0.409

## Data Availability

The data used to support the findings of this study are included within the article.

## References

[1] Salar O., El-Sharkawy A. M., Singh R., Speake W. (2013). Internal hernias: a brief review. *Hernia*.

[2] Murphy K. P., O'Connor O. J., Maher M. M. (2014). Adult abdominal hernias. *American Journal of Roentgenology*.

[3] Tepeš M., Kirac I., Glavan E., Doko M. (2015). Internal Hernias in Acute Abdomen: Review of Literature and Report of four Cases. *Collegium Antropologicum*.

[4] Taniguchi K., Iida R., Ota K., Asakuma M., Uchiyama K., Takasu A. (2018). Single-port surgery (SPS) strategy for small bowel obstruction (SBO) caused by postoperative internal hernia. *Medicine*.

[5] Saklani A., Naguib N., Tanner N., Moorhouse S., Davies C. E., Masoud A. G. (2012). Internal herniation following laparoscopic left hemicolectomy: An underreported event. *Journal of Laparoendoscopic & Advanced Surgical Techniques*.

[6] Mukherjee K., Wise P. E. (2013). Internal hernia through the gastrohepatic ligament after laparoscopic restorative proctocolectomy. *The American Surgeon*.

[7] Masubuchi S., Okuda J., Tanaka K. (2013). An internal hernia projecting through a mesenteric defect following laparoscopic-assisted partial resection of the transverse colon to the lesser omental cleft: Report of a case. *Surgery Today*.

[8] Yoshida T., Kinugasa T., Oka Y. (2014). Bowel obstruction caused by an internal hernia that developed after laparoscopic subtotal colectomy: a case report. *Journal of Medical Case Reports*.

[9] Daskalaki A., Kaimasidis G., Xenaki S., Athanasakis E., Chalkiadakis G. (2015). Internal-mesocolic hernia after laparoscopic left colectomy report of case with late manifestation. *International Journal of Surgery Case Reports*.

[10] Cabot J. C., Lee S. A., Yoo J., Nasar A., Whelan R. L., Feingold D. L. (2010). Long-term consequences of not closing the mesenteric defect after laparoscopic right colectomy. *Diseases of the Colon & Rectum*.

[11] Toh J. W. T., Lim R., Keshava A., Rickard M. J. F. X. (2016). The risk of internal hernia or volvulus after laparoscopic colorectal surgery: a systematic review. *Colorectal Disease*.

[12] LaMattina J. C., Powell J. M., Costa N. A. (2017). Surgical complications of laparoendoscopic single-site donor nephrectomy: a retrospective study. *Transplant International*.

[13] Hosono S., Ohtani H., Arimoto Y., Kanamiya Y. (2007). Internal hernia with strangulation through a mesenteric defect after laparoscopy-assisted transverse colectomy: Report of a case. *Surgery Today*.

[14] Jimi S.-I., Hotokezaka M., Eto T.-A. (2007). Internal herniation through the mesenteric opening after laparoscopy-assisted right colectomy: Report of a case. *Surgical Laparoscopy Endoscopy & Percutaneous Techniques*.

[15] Sadatomo A., Miyakura Y., Zuiki T. (2013). Sigmoid volvulus after laparoscopic surgery for sigmoid colon cancer.. *Asian Journal of Endoscopic Surgery*.

[16] Izumi J., Satoh K., Iwasaki W., Miura T., Fujimori S. (2017). Small bowel obstruction caused by the ingestion of a wooden toothpick: The ct findings and a literature review. *Internal Medicine*.

[17] Santangelo M. L., Grifasi C., Criscitiello C. (2017). Bowel obstruction and peritoneal carcinomatosis in the elderly. A systematic review. *Aging Clinical and Experimental Research*.

[18] Lee S. Y., Kim C. H., Kim Y. J., Kim H. R. (2017). Internal hernia following laparoscopic colorectal surgery: a rare but fatal complication. *Hernia*.

[19] Kim C. H., Joo J. K., Kim H. R., Kim Y. J. (2014). The incidence and risk of early postoperative small bowel obstruction after laparoscopic resection for colorectal cancer. *Journal of Laparoendoscopic & Advanced Surgical Techniques*.

[20] Sugiyama M., Sakaguchi Y., Oki E. (2016). Clinical Significance of Closure of Mesenteric Defects in Laparoscopic Colectomy. *Surgical Laparoscopy, Endoscopy & Percutaneous Techniques*.

[21] Abarca F., Saclarides T. J., Brand M. I. (2011). Laparoscopic colectomy: Complications causing reoperation or emergency room/hospital readmissions. *The American Surgeon*.

